# Technological Innovations in Surgical Approach for Thyroid Cancer

**DOI:** 10.1155/2010/490719

**Published:** 2010-07-27

**Authors:** Brian Hung-Hin Lang, Chung-Yau Lo

**Affiliations:** Division of Endocrine Surgery, Department of Surgery, University of Hong Kong Medical Centre, Queen Mary Hospital, 102 Pokfulam Road, Hong Kong

## Abstract

Over the last decade, surgeons have witnessed dramatic changes in surgical practice as a result of the introduction of new technological advancement. Some of these changes include refinement of techniques in thyroid cancer surgery. The development of various endoscopic thyroidectomy techniques, the addition of the da Vinci robot, and the use of operative adjuncts in thyroid surgery, such as intraoperative neuromonitoring and quick intraoperative parathyroid hormone, have made thyroid cancer surgery not only safer and better accepted by patients with thyroid cancer but also offer them more surgical treatment options.

## 1. Introduction

New technologies have had a positive impact on our ability to diagnose and treat many surgical conditions [1]. Over the last decade, surgeons have witnessed dramatic changes in surgical practice as a result of the introduction of new technologies or technological advancement. Thyroid cancer is the commonest endocrine-related tumor. In our locality, its age-adjusted incidence has doubled over the last 25 years, and a similar trend has been reported elsewhere [2]. New technologies have had important influence in the management of this disease. In addition to improving the preoperative diagnostic accuracy and cancer staging with various imaging modalities, the techniques of thyroid cancer surgery have been refined and evolved in this era of technological advancement. In applying these new technologies, it is believed that surgical morbidity can be further reduced, hospital stay shortened, and patient satisfaction enhanced [3]. The present paper aimed at evaluating how some of these new technological innovations might improve patient outcomes and offer new surgical treatment options for patients diagnosed with thyroid cancer. These innovations include the development of various endoscopic thyroidectomy techniques, the addition of the da Vinci robot surgical system, as well as the use of operative adjuncts such as intraoperative neuromonitoring (IONM) and quick intraoperative parathyroid hormone (IOPTH).

## 2. Endoscopic Thyroidectomy

The application of endoscopic visualization to thyroid surgery has allowed surgeons to perform thyroidectomy through incisions far smaller and less visible than the conventional Kocher's incision—the so-called “less is more.” In general, these endoscopic techniques attempt to minimizing the extent of dissection, improving cosmesis, reducing postoperative pain, shortening hospital stay, and enhancing postoperative recovery. Michel Gagner was the first to apply endoscopic technique to neck surgery when he reported a totally endoscopic subtotal parathyroidectomy for a 37-year-old man suffering from familial hyperparathyroidism [4]. Although the endoscopic procedure took over 5 hours, it demonstrated the technical feasibility and safety. Over the turn of the last century, an increasing number of different endoscopic techniques have been described and may be categorized into namely cervical or direct and extracervical or indirect approaches [5]. The former is considered as truly minimally invasive since the skin incisions are small in the neck with direct access to the thyroid gland. On the other hand, the extracervical approach is considered as an endoscopic instead of minimally invasive approach because incisions are made distant from the neck and so the procedure requires more extensive tissue dissections [6]. However, despite its invasiveness, it offers superior early cosmetic outcome because potentially unsightly scars can be hidden. This approach has been adopted more often in Asian countries where cosmesis seems to be of greater concern.

### 2.1. Cervical/Direct Approaches

These approaches include the endoscopic lateral cervical approach and the minimally invasive video-assisted thyroidectomy (MIVAT). In the endoscopic lateral cervical approach, two 2.5 mm and one 10 mm trocars are inserted under direct vision along the anterior border of the sternocleidomastoid muscle on the side of resection. Using endoscopic instruments, the dissection starts from the lateral aspect of the thyroid gland and moves medially with identification of the recurrent laryngeal nerve (RLN), parathyroid glands and skeletonisation of the superior and inferior thyroid vessels [7]. Excellent visualization of RLN and parathyroid glands is possible with magnification by the endoscope. However, this technique is limited to unilateral thyroid resection and its application in thyroid cancer surgery is restricted to subcentimeter papillary thyroid carcinoma (PTC) detected by high-resolution ultrasound machines. In contrast, the MIVAT would be preferred if bilateral thyroid resection is required because the incision is made in the middle instead of the lateral aspect of the neck. A 1.5 cm incision is made in the middle of the neck about 2 cm above the sternal notch. Blunt dissection is then carried out to separate the strap muscle from underlying thyroid lobe. A 5 mm 30 degree endoscope is placed inside the 1.5 cm wound for lighting and visualization. The procedure is performed under endoscopic view with the operating space maintained by external retraction. This technique was first applied for selected benign thyroid conditions by Miccoli et al. in 2000 [8]. However, with improvement in techniques, MIVAT has become increasingly adopted for low-to-intermediate risk differentiated thyroid cancer [9]. MIVAT has been shown to achieve similar completeness of resection [10, 11] and 5-year survival outcomes as those with low and intermediate risk PTC undergoing conventional thyroidectomy [9]. In addition, it has been shown that a concomitant central neck dissection is technically feasible in MIVAT during initial total thyroidectomy [12]. Also, for patients with low risk PTC with concomitant lateral lymph node metastases, a minimally invasive video-assisted functional lateral neck dissection through a small neck incision is also technically possible [13].

### 2.2. Extracervical/Indirect Endoscopic Approaches

Unlike the cervical approaches, these approaches involve making incisions either in the chest, breast, and/or axilla to hide the scars with clothing [14]. Ikede et al. first described these approaches by placing three ports in the axilla with low-pressure gas insufflation for maintaining the operating space. Although cosmetic results were excellent, the procedure was technically demanding and time consuming because of unintentional easy gas leakage and frequent interference of the 3 operating surgical instruments in the small available space in the axilla [15]. Kang et al. modified this technique by making this approach gasless with the space maintained by a specially designed skin-lifting external retractor [16]. In this approach, the procedure began with a 4 cm to 5 cm incision in the axilla and then a subcutaneous space was created from the axilla to the thyroid gland. To avoid the problem of interference of instruments, an additional 5 mm port was inserted in the chest area for medial retraction of the thyroid gland. Kang et al. recently reported their experience with this approach after performing 581 cases [16]. Among these patients, 410 patients had low-risk PTC. In their series, concomitant central neck dissection was performed and the rate of lymph node metastasis was 27.3% [16].

To further increase the degree of angulations and freedom of interference between instruments, a combined axillo-breast approach was developed utilizing 2 circumareolar trocars in the breast and a single trocar in the ipsilateral axilla. This approach was later modified by using bilateral axillary ports to allow better exposure to both sides of the thyroid compartment. This approach is now known as the bilateral axillo-breast approach (BABA). Despite the extensive tissue dissection, when compared with the conventional open approach, BABA has been shown to have similar results in terms of transient hypocalcemia, bleeding, permanent RLN paralysis and length of hospital stay [17]. More recently, a Korean group tried to eliminate wounds around the chest or breast areas all together by making incisions in the axilla and postauricular areas instead. They reported a small series of 10 patients using this approach and 7 underwent bilateral thyroid resection for low-risk PTC. They demonstrated the feasibility of this technique of scarless (in the neck) thyroid surgery [18].

## 3. Robotic-Assisted Thyroidectomy

The application and feasibility of the endoscopic approach was given a further boost with the availability of various robotic systems such as the da Vinci system (Intuitive Surgical, Sunnyvale, California). Unlike other cancers such as prostate cancer, the initial enthusiasm of using the robot in thyroid cancers was not great because of its relatively high cost, bulkiness of the robotic arm, and long operating time. However, since the publication of two large surgical series demonstrating the feasibility and safety of robotic-assisted thyroidectomy in differentiated thyroid carcinoma, an increasing number of specialized surgical centers worldwide are beginning to accept and perform this procedure. The theoretical advantages of using the robot over the endoscopic approach include the three-dimensional view offer to the operating surgeon, the flexible robotic instruments with seven degree of freedom and 90° articulation, the increased tactile sensation, and the ability to filter any hand tremors [19]. Kang et al. recently reported their experience of 200 robot-assisted total thyroidectomy using the gasless transaxillary approach for low-risk PTC with concomitant central neck dissection and found excellent short-term results in terms of postoperative pain and patients' satisfaction [20]. This was followed briefly by another report of 338 benign and malignant cases using the same transaxillary [21]. To date, this group has performed over 1000 cases. A separate Korean group also reported similar results using the da Vinci robot via the BABA technique [22]. Although both techniques have been demonstrated to be feasible and safe, they have been limited to a few high-volume specialized centers. The surgeons performing these operations have had years of operating experience with the endoscopic approach and so the learning curve for a novel, nonendoscopic thyroid surgeon or someone who predominantly perform open thyroid procedures, remains undefined but is likely to be longer than one might think. Furthermore, better comparative studies such as a randomized controlled trial between robotic-assisted and endoscopic thyroidectomy are needed in order to better assess the added patient outcome benefits over the latter approach.

## 4. Surgical Adjunct: IONM

RLN injury is a leading cause of litigation in thyroid surgery [23]. To those with this injury, it not only affects the voice quality but also diminishes the overall quality of life because of communication, social and work-related problems [24]. Routine RLN identification is currently the gold standard of care in thyroid surgery. However, with the availability of IONM, the issues are whether this new technology could further enhance RLN preservation and reduce the risk of iatrogenic RLN injury in thyroid surgery or thyroid cancer surgery in particular. 

Although IONM has been around for over 3 decades, its widespread usage in the surgical practice only dates back to 5–10 years. There has been an increased interest in applying this technique for thyroid surgery because of the introduction of new and user-friendly devices from technological advance [25]. Currently, there are two types of IONM systems, namely, those with electromyographic (EMG) documentation and those without EMG documentation. The former involves RLN stimulation with registration of the elicited laryngeal muscle activity through endoscopic insertion of electrodes into the vocal fold or with the use of endotracheal surface electrodes. The latter utilizes RLN stimulation with observation of posterior cricoarythenoid muscle contraction or palpation or intraoperative inspection of vocal cord function [26, 27]. To date, there is no consensus on which is the best system, and the choice depends on the availability of which system in your institution and the operator familiarity or experience. Regardless of which systems, there are potential flaws and pitfalls. In general, the positive predictive value (PPV) is proportionally low with this technology. That means that when a nerve has no signal during stimulation, it does not mean that it is injured. In fact, in our experience, the PPV was only 15% in low-risk thyroid surgery, that is, approximately only 1 out of 9 RLNs with no signals had an actual injury. This might be due to some technical errors such as detachment or displacement of electrodes or poor contact of the probe with the nerve due to inadequate exposure [28]. Perhaps, direct vagal stimulation could possibly reduce some of these errors but need more unnecessary dissection. Even more intriguing is the fact that this technique is also associated with false negative results, albeit rarely. In our experience, among 271 nerves at risk, 15 (5.5%) ended with RLN palsy but of these, 7 still had a positive IONM signals. Therefore, it seems that IONM might not be able to detect “sublethal” injury to RLN. It is possible that the action potential could be propagated along the neural pathway, as detected by the IONM, but not to the extent of initiating laryngeal muscle contraction during the postoperative period [25, 28]. Fortunately, all these injuries would invariably recover.

On the other hand, although the objective of the use of this device is to avoid RLN injury during thyroid surgery, the evidence of supporting its routine use has been weak. The first multicenter study including 29,998 RLNs at risk confirmed that the incidence of RLN palsy was not significantly reduced by the additional use of IONM when routine RLN identification was performed [27]. There were more than 20 publications addressing this issue but majority of these studies were heterogeneous in terms of patients' characteristics (such as primary operations versus reoperations or benign versus malignant goiters), IONM techniques and the extent of resection (i.e., total versus subtotal lobectomy). A recent literature review could not definitely draw confirm conclusions or evidence on the effectiveness of IONM in reducing RLN injury in thyroid surgery [26]. Furthermore, most studies were either case-series with no control group or retrospective studies with inadequate statistical power to demonstrate a difference between those with or without using IONM. In fact, a randomized study utilizing approximately 7,000 patients in each arm of patients undergoing thyroidectomy with or without IONM will be required to have adequate statistical power to show a difference in outcome with reference to RLN paralysis [26, 27]. Interestingly though, the first prospective randomized study comparing IONM with routine RLN visualization only was recently published [28]. In this study, approximately 500 patients were randomized into each arm. The number of patients recruited in each arm was based on the principle of detecting a 2% difference in the incidence of transient RLN injury with a 90% probability at *P* < .05. This study did demonstrate a statistically significant difference in reducing transient RLN injury when IONM was adopted in comparison with RLN visualization only. However, as expected, the rate of permanent RLN injury was similar in the two study arms because of inadequate statistical power. Nevertheless, despite the inadequate power of most published IONM studies, there seemed to be a trend toward improved RLN protection with the use of this new technology [26]. In addition, the IONM may be of potential benefit for “difficult” cases such as reoperative thyroidectomy, locally advanced thyroid cancers or central neck dissection for cancer recurrence. Perhaps, for the novel and relatively inexperienced surgeons, the IONM might prove to be extremely invaluable for these difficult cases.

## 5. Surgical Adjunct: IOPTH or Quick Intraoperative Parathyroid Assay (qPTH) as an Assessment of Posthyroidectomy Hypoparathyroidism

Hypoparathyroidism is a common complication after bilateral thyroid resections or total thyroidectomy. Up to 30% of patients after total thyroidectomy develop temporary hypoparathyroidism [29]. There are many identifiable risk factors leading to postoperative hypoparathyroidism including thyroidectomy for thyrotoxicosis and thyroid cancer, thyroid reoperations, reduced stores of vitamin D, increased extent of thyroid resection, and need of concomitant central neck dissection [30, 31]. Patients undergoing thyroidectomy for thyroid cancer are particularly prone to hypoparathyroidism because they often need a more complete thyroid resection together with neck dissection. In fact, total thyroidectomy and routine concomitant central neck dissection has now been increasingly practiced worldwide for almost types of well-differentiated thyroid cancer to achieve lower recurrences, better disease-free survival, and enhanced postoperative athyroglobulinemia [32]. However, it has been shown that up to 60% of patients after concomitant central neck dissection could develop transient hypocalcemia secondary to the frequent occurrence of unintentional or incidental parathyroidectomy [33]. Therefore, in the presence of such a high incidence of postoperative hypoparathyroidism, the need of routine postoperative inpatient calcium monitoring remains questionable after thyroid cancer surgery while the early routine administration of oral calcium and/or vitamin D supplements seems to be relevant and can facilitate the early discharge from hospital shortly after surgery without developing unpleasant hypocalcemic symptoms [34]. In fact, a recent randomized study supported this strategy because routine administration of oral calcium was shown to markedly reduce the severity and symptoms of hypocalcemia [35]. However, the adoption of this strategy could lead to overtreatment in patients who do not have hypocalcaemia leading to rebound hypercalcemia and increased medication costs. On the other hand, this strategy might lead to inadequate treatment in patients with severe symptomatic hypocalcaemia as oral calcium alone may not fully correct the hypocalcemia and so vitamin D supplements is indicated in such situation [36].

On the other hand, inpatient serial close monitoring of serum calcium is recommended after total thyroidectomy because most symptomatic hypocalcemia occurs around 24–28 hours after surgery [37]. A 24-hour or longer hospital stay is invariably required. Therefore, efforts are made to shorten hospital stays, decrease biochemical blood tests, and reduce hospital costs by adopting other strategies to achieve early prediction of postthyroidectomy hypocalcemia. With the availability of IOPTH and wide application in patients undergoing minimally invasive parathyroidectomy to predict postoperative cure, this new surgical adjunct has been applied to thyroid surgery to monitor parathyroid function and to predict the occurrence of postoperative normocalcaemia or hypocalcaemia. In our early prospective study of using IOPTH in predicting hypocalcemia in 100 consecutive patients (including 33 patients with differentiated thyroid cancer) who underwent either total or completion thyroidectomy, we found that a normal level of IOPTH at 10 mins or a level less than 75% decline in IOPTH at 10 mins after excision of thyroid gland accurately identified normocalcemia [38]. It was suggested that intraoperative or early postoperative parathyroid hormone assay might be a sensitive tool to confirm postoperative normocalcaemia and identify patients atrisk of developing postoperative hypocalcaemia. Since then, up to 30 different investigators have published their results of using various different IOPTH assays in predicting hypocalcemia after total thyroidectomy. The IOPTH levels and their rate of decline at various time points after surgery could be utilized for prediction of postoperative hypocalcaemia with variable sensitivity, specificity, and accuracy [39, 40]. However, based on two evidence-based reviews, it was recommended that the IOPTH level within a few hours after thyroid surgery could accurately predict postoperative normocalcaemia and identify patients at-risk of developing hypocalcemia, particularly severe, symptomatic hypocalcemia [34, 41]. It was suggested that patients could be stratified into high- or low-risk groups and PTH should be measured at 1–6 hrs after operation in comparison to preoperative PTH. A < or > 65% decline at 6 hours after operation should allow early discharge or facilitate the decision of early calcium supplement. On the other hand, a strategy of 2 cut-off points should be considered with a high accuracy. A <50% decline within few hours after surgery allowed early discharge while a >90% decline necessitated early calcium supplement because of the accuracy in predicting normocalcaemia and hypocalcaemia, respectively [41]. For those patients with 50%–90% decline, either serial calcium monitoring or routine treatment should be considered. In the AES guideline, one single serum PTH measurement is recommended at 4 hrs after operation [42]. A normal PTH can predict normocalcaemia, and patients can be discharged early with 7% subsequently developing mild hypocalcaemia. For patients with undetectable PTH level, oral calcium and vitamin D analogue should be administered early to avoid symptomatic hypocalcaemia. Intermediate or subnormal PTH level is a less accurate predictor of hypocalcaemia. In that case, oral calcium should commence or patients should be monitored with serial calcium levels for the need of calcium and/or vitamin D analogue [42]. Therefore, PTH assay can now be considered as a perioperative adjunct to predict normocalcaemia or hypocalcaemia with reasonable accuracy. It can facilitate early discharge, avoid routine calcium replacement, facilitate early calcium replacement to avoid symptomatic hypocalcaemia and decrease overall cost as well as increase patients' satisfaction.

## 6. Conclusions

New technologies have undoubtedly had a positive impact on the surgical management of thyroid cancer. The application of endoscopic visualization of the thyroid gland has allowed surgeons to perform safe surgery from extracervical skin incisions. Although short-term outcome studies in various endoscopic techniques demonstrated comparative results as conventional open thyroidectomy for differentiated thyroid cancer, this particular operative approach is indicated for selected patients only and the benefit is still considered marginal with concerns of higher associated cost and longer operating time in performing these procedures. Although the robot procedures might offer some theoretical advantages over the endoscopic procedures, better-designed prospective comparative studies are required. Despite the lack of strong evidence for the benefit of routine use of IONM, there is a trend toward improved RLN protection and reduced iatrogenic RLN injury. As a surgical adjunct, IOPTH is being actively sought as a cost-effective tool for predicting postoperative hypocalcaemia in patients undergoing total thyroidectomy for thyroid cancer.
